# Genome-wide transcriptomics of aging in the rotifer *Brachionus manjavacas*, an emerging model system

**DOI:** 10.1186/s12864-017-3540-x

**Published:** 2017-03-01

**Authors:** Kristin E. Gribble, David B. Mark Welch

**Affiliations:** Josephine Bay Paul Center for Comparative Molecular Biology and Evolution, Marine Biological Laboratory, Woods Hole, MA 02543 USA

**Keywords:** Aging, Rotifer, Monogonont, RNA-Seq, Transcriptome

## Abstract

**Background:**

Understanding gene expression changes over lifespan in diverse animal species will lead to insights to conserved processes in the biology of aging and allow development of interventions to improve health. Rotifers are small aquatic invertebrates that have been used in aging studies for nearly 100 years and are now re-emerging as a modern model system. To provide a baseline to evaluate genetic responses to interventions that change health throughout lifespan and a framework for new hypotheses about the molecular genetic mechanisms of aging, we examined the transcriptome of an asexual female lineage of the rotifer *Brachionus manjavacas* at five life stages: eggs, neonates, and early-, late-, and post-reproductive adults.

**Results:**

There are widespread shifts in gene expression over the lifespan of *B. manjavacas*; the largest change occurs between neonates and early reproductive adults and is characterized by down-regulation of developmental genes and up-regulation of genes involved in reproduction. The expression profile of post-reproductive adults was distinct from that of other life stages. While few genes were significantly differentially expressed in the late- to post-reproductive transition, gene set enrichment analysis revealed multiple down-regulated pathways in metabolism, maintenance and repair, and proteostasis, united by genes involved in mitochondrial function and oxidative phosphorylation.

**Conclusions:**

This study provides the first examination of changes in gene expression over lifespan in rotifers. We detected differential expression of many genes with human orthologs that are absent in *Drosophila* and *C. elegans,* highlighting the potential of the rotifer model in aging studies. Our findings suggest that small but coordinated changes in expression of many genes in pathways that integrate diverse functions drive the aging process. The observation of simultaneous declines in expression of genes in multiple pathways may have consequences for health and longevity not detected by single- or multi-gene knockdown in otherwise healthy animals. Investigation of subtle but genome-wide change in these pathways during aging is an important area for future study.

**Electronic supplementary material:**

The online version of this article (doi:10.1186/s12864-017-3540-x) contains supplementary material, which is available to authorized users.

## Background

Aging is a complex process characterized by the progressive degeneration of a healthy phenotype and correlated with a decline in the ability to withstand cellular stress and damage. The subject of investigation for decades, the underlying molecular genetic causes of and responses to aging remain an area of active study. Research from model systems has characterized a range of physiological and molecular phenotypes associated with aging. These include genomic instability caused by accumulation of DNA damage, dysregulation of repair mechanisms, and telomere attrition; epigenetic alterations; dysregulation of transcription; loss of proteostasis; cellular senescence; and deregulated nutrient sensing, metabolic pathways, and energy use (reviewed in [1]). Separating causation from correlation between these phenotypes and aging remains a challenge, however.

Many of the genes and gene networks that modulate aging are conserved across animal phyla. For this reason, the highly tractable model systems *Drosophila* and *Caenorhabditis* have provided fundamental advances in our understanding of the genetic control of cellular processes that affect aging. There is a growing realization that increasing the evolutionary breadth in animal systems used in aging studies will lead to discovery of effects and mechanisms that are more likely to be robust and reveal fundamental principles of aging. The use of diverse models may also reveal previously unknown genetic factors involved in healthy aging in humans. The lineages leading to *Drosophila melanogaster* and *Caenorhabditis elegans* have each undergone significant genome reduction, and these standard model systems lack many vertebrate gene homologs that are present in other invertebrates [2–9]. In addition, arthropods and nematodes are more closely related to each other than originally thought [10, 11], limiting the evolutionary range in comparative studies of aging [12] and thus the degree to which conclusions can be reliably generalized from these models to humans.

Rotifers are small aquatic invertebrates that have been used in aging studies for nearly 100 years but have only recently been developed as a modern model system for the study of aging [13, 14]. They are early-branching triploblast animals that have not undergone extensive genome reduction, but instead share many genes with vertebrates that are missing in flies and worms [15, 16]. This suggests that the rotifer genome may contain genes and pathways that modulate aging in vertebrates but are not present in other invertebrate model systems.

Rotifers develop directly without a larval stage and are eutelic, without cell division after hatching except in the germline [17]. Many species can be easily cultured in water on a simple diet of bacteria or single-celled algae, and even a large experiment can be conducted in a small number of tissue culture dishes. Most have an asexual stage to their life cycle, and can be cultured asexually indefinitely, maintaining genetic identity across generations and between test conditions.

The rotifer *Brachionus manjavacas* has a well-characterized life cycle and aging phenotype. Under standard laboratory conditions, asexual *B. manjavacas* neonates increase in size quickly after hatching and produce their first offspring within 48 h. Reproduction increases to a maximum of 6 offspring/day around day 5, then declines to the end of the reproductive period on approximately day 8; the post-reproductive (senescent) period last for another 2–4 days. As a *B. manjavacas* female ages, her color changes from translucent to opaque, her foot begins to drag instead of being tucked up against the body, she cannot attach as firmly to a substrate, and swimming speed declines dramatically [14, 18]. Median lifespan is approximately 10 days; death is characterized by a lack of motion of cilia, appendages, or internal organs and is frequently accompanied by a loss of membrane integrity.

**Fig. 1:**
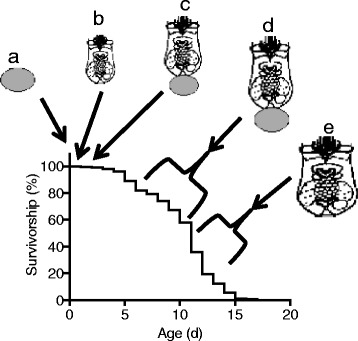
Representative survivorship curve of asexual *Brachionus manjavacas* females. The life stages collected for analysis are shown: **a** eggs; **b** neonates, 3 hrs old; **c** early reproduction, 36 h old; **d** late reproduction, 5–7 days old; **e** post-reproductive, 8–9 days old. Median rotifer lifespan is 9–11 days. Reproduction begins at 36 h and peaks by day 5; rotifers are post-reproductive after 8–9 days, with a post-reproductive period of 2–4 days

*Brachionus* rotifers have recently been used to study the effects on healthspan and aging of stress [18–21]; temperature [22]; and dietary restriction and metabolism, including maternal effects [23–28]. To define the transcriptional changes associated with normal aging in this emerging model system, we conducted RNA-Seq at five timepoints over the lifespan of *B. manjavacas*, from eggs to senescence (Fig. 1). This study provides the first insights into genome-wide changes in gene expression over the lifespan of a rotifer, and continues development of this model system to study the biology of aging.

## Methods

### Culture

We maintained the monogonont rotifer *Brachionus manjavacas* in batch culture in 15 ppt Instant Ocean on a diet of the chlorophyte alga *Tetraselmis sueccica*. We grew *T. sueccica* in bubbled f/2 medium [29] prepared in 15 ppt Instant Ocean. We maintained both *B. manjavacas* and *T. suecica* at 21 °C on a light:dark cycle of 12 h:12 h.

### Sample preparation and sequencing

One week prior to beginning the experiments, we inoculated a new batch culture of *B. manjavacas* at approximately 1 rotifer/25 mL. To prevent induction of sexual reproduction due to crowding, we maintained the culture at a density below 1 rotifer/2 ml by daily sieving and transfer into new *T. suecica* and medium. Concentrations of *T. suecica* were always above 6 x 10^5^ cells/ml, which we previously demonstrated allows feeding *ad libitum* [28].

We collected samples at five time points over the lifespan of amictic *B. manjavacas* females (Fig. 1): eggs, neonates 1–3 h old, 36 h old early reproductive females, reproductive females of mixed ages from 3 to 6 d old, and post-reproductive females between 6 and 9 days old. Reproductive females carry one or more eggs, which we removed prior to harvesting by vortexing. For each time point, we harvested 100–200 rotifers into a 1.5 ml centrifuge tube, immobilized them using distilled water and centrifuged to a pellet. We resuspended the pellet in 150 μl TriZOL reagent (Invitrogen, Waltham MA), mashed it with a pestle, brought the sample up to 1 mL with TriZOL reagent, and froze at − 80 °C until RNA extraction.

We constructed RNA-Seq libraries starting with total RNA extracted using the TriZOL reagent protocol, followed by removal of residual DNA using Turbo DNA-free (Ambion, Waltham, MA). We used the Ovation 3’-DGE kit (NuGEN, San Carlos, CA) to create cDNA from mRNA using linear amplification to prevent PCR bias, sheared to 400–500 bp with Covaris, end-repaired fragments (S1 Nuclease, Promega, Madison, WI and Quick Blunting Kit, New England Biolabs, Ipswich, MA), and adenylated 5’ ends using Taq (New England Biolabs). We ligated unique TruSeq compatible indexed adapters to each library and size selected to 400–600 bp using Pippin Prep (Sage Science, Beverly, MA). Illumina HiSeq sequencing generated an average of 40 million 2x100base read pairs.

We repeated the entire procedure from inoculation through sequencing for a biological replicate set of libraries, which generated an average of 34 million pairs of 100 base reads.

### Transcriptome assembly and annotation

We filtered reads according to Minoche [30] and mapped high quality read pairs to our published *B. manjavacas* transcriptome assembled from 454 data (SRR801079; [31]) using CLC Genomics Workbench 7 (https://www.qiagenbioinformatics.com/). We then assembled the unmapped reads and unassembled 454 reads and added these contigs to the assembly. To remove transcripts from media contaminants and *T. suecica*, we eliminated contigs that had a top BLAST hit in the NCBI refseq protein database to a non-Metazoan with an *E* value less than 10^−6^, or that had no hit with an *E* value less than 10^1^; 43,149 contigs remained. We annotated these contigs by BLAST to the KEGG, Swissprot, and Human Protein Reference databases at an *E* value cutoff of 10^−10^; we used the top hits to HPRDv9 to assign HGNC gene symbols for gene set enrichment analysis, described below. We refer to genes by HGNC gene symbol whenever possible. We used reciprocal best blastx scores to assign orthology between each transcript and all peptides from *Homo sapiens* (assembly GRCh38.p7), *Drosophila melanogaster* (assembly BDGP5), and *Caenorhabditis elegans* (assembly WBcel235) downloaded from www.ensembl.org. We considered a gene to be present in humans and *B. manjavacas* and absent in flies and worms if the *E* value between human and rotifer was less than 10^−20^ and greater than 10^−10^ to flies or worms.

### Differential gene expression and pathway analysis

We mapped quality filtered reads separately from each library to the assembly using CLC. To prevent bias toward transcripts with very low levels of expression, we confined our differential expression analysis to the 22,064 contigs with an average FPKM ≥ 1 across all five ages and two replicates. The mean length of these contigs was 906 bp, with an N50 of 1231 bp. We determined significantly differently expressed transcripts between each life stage using baySeq [32], with an FDR cutoff of 0.05 (see Additional file 1 for baySeq R scripts). These composed the test set to find enriched Gene Ontology (GO) terms using Blast2GO. Refseq annotation was imported for the entire transcriptome of 22,064 contigs, and additional annotation with GO IDs and InterPro identifiers was conducted in Blast2GO with a permissive evalue cutoff of 10^−3^. Up- and down-regulated transcripts for each life stage transition were analyzed separately with all 22,064 contigs as the reference set for the Fisher’s exact test (FDR ≤ 0.05, two-tailed analysis). Significantly differently expressed GO terms were reduced to specific terms for analysis.

We conducted gene set enrichment analysis (GSEA; [33, 34]) on pairwise consecutive time points. For input to GSEA, we annotated the *B. manjavacas* transcriptome with HGNC gene names as described above [35]. Transcripts with the same HUGO annotation were summed for analysis, and a constant of ten was added to all FPKM values to avoid bias toward genes with low expression when converting to log2. We searched for enrichment of KEGG pathways gene sets using our entire expression dataset, with 1000 permutations by gene set. The metric chosen for ranking genes was the log2 ratio of classes; significant enrichment, either up or down, was designated as FDR ≤ 0.25. The normalized enrichment statistic was used to create a heat map of KEGG pathways. Leading edge analysis in GSEA was used to identify genes shared among more than one enriched gene set; genes that are differentially expressed at a given age and are found in several pathways are more likely to have an impact on multiple functions than are those found in only a single pathway.

For a detailed look at gene expression in pathways of interest, KEGG pathway annotation was used to select relevant transcripts, and these were clustered according to expression level with the hierarchical cluster module of GenePattern [36] using Pearson correlation of log2 transformed FPKM values.

## Results and discussion

**Fig. 2:**
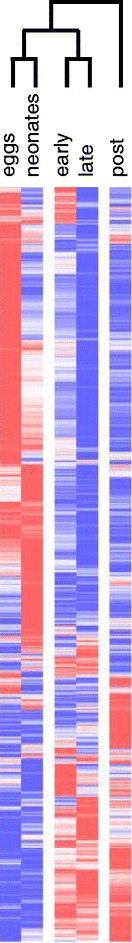
Relative expression profiles between life stages in *Brachionus manjavacas.* Heatmap of genes significantly differentially expressed between at least two life stages with hierarchical clustering of average FPKM across two biological replicates performed by average linkage 1- Pearson’s correlation. Data are row-normalized, with *red* indicating highest expression and *blue* indicating lowest expression

**Fig. 3:**
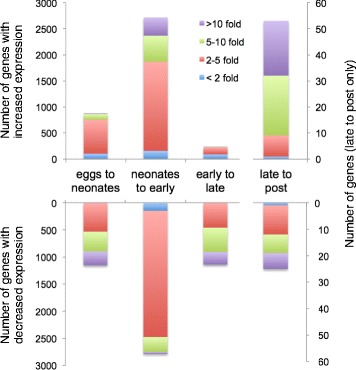
Differential expression at life stage transitions in *Brachionus manjavacas.* Bars indicate the number of significantly differentially expressed genes is indicated for each transition, with color indicating fold-change (3-fold change = 2^3^ difference in transcript abundance). Note that the late- to post-reproductive transition uses the right y-axis

**Fig. 4:**
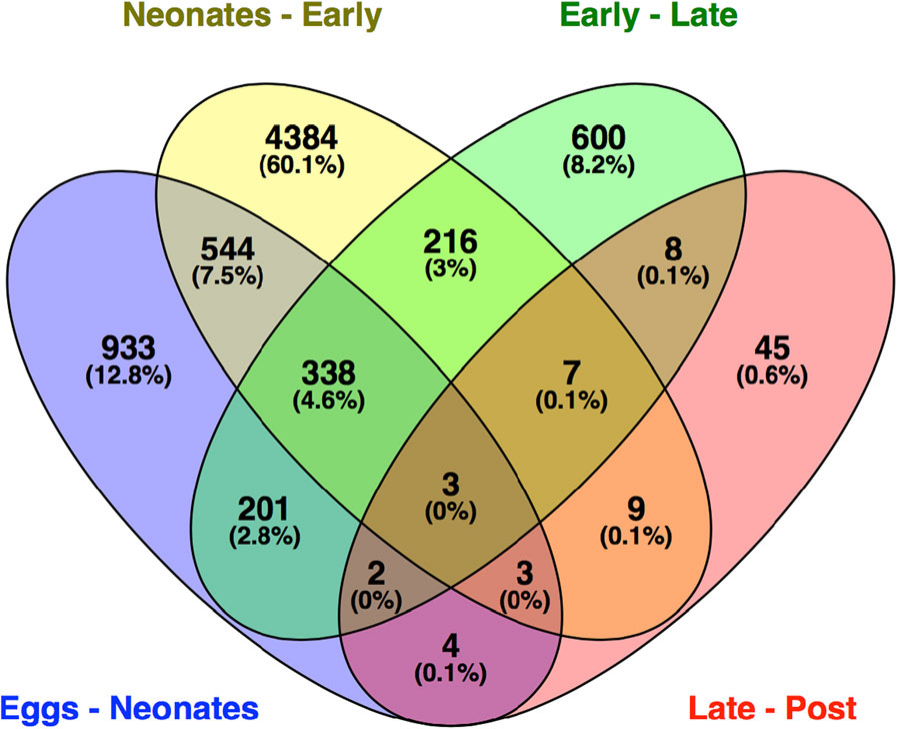
Shared differential expression between life stage transitions in *Brachionus manjavacas.* Venn diagram showing number and percentage of significantly differentially expressed genes (both up and down-regulated), in each transition and the overlap of genes shared between transitions

Aging in *B. manjavacas* is characterized by large shifts in transcript abundance between major life stages (Table 1, Figs 2, 3 and 4), which we take as a first-order proxy for gene expression. Among the 22,064 contigs representing genes with an average FPKM > 1, 15,826 genes had significant differential expression in one or more pairwise comparisons of life stages (FDR < 0.05). Expression profiles were similar between eggs and neonates, and between early and late reproductive stages; these stages broadly represent developmental and reproductive periods, respectively. The expression profile of the post-reproductive or senescent stage was distinct from the four other life stages.

**Table 1:** The number of genes significantly differentially expressed between each life stage (baySeq, FDR < 0.05)

	Eggs		Neonates		Early		Late	
	Up	Down	Up	Down	Up	Down	Up	Down
Neonates	1152	876	-	-	-	-	-	-
Early	3505	3469	2786	2718	-	-	-	-
Late	6945	5814	4895	3740	1140	236	-	-
Post	5266	4557	657	844	550	350	25	53

**Fig. 5:**
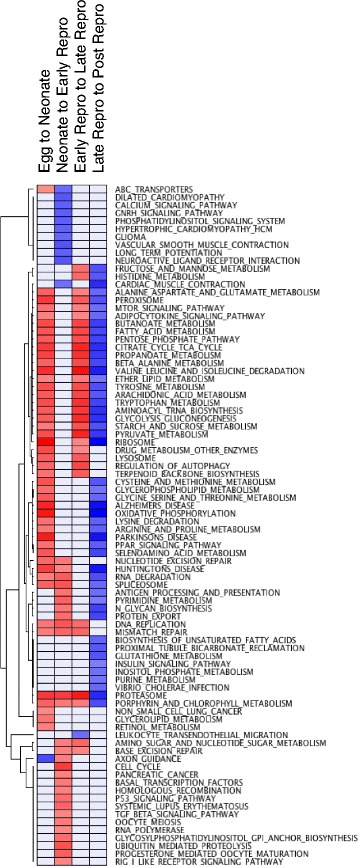
Heatmap of KEGG pathways significantly enriched between life stages in *Brachionus manjavacas*. Pathways that were significantly up-regulated or down-regulated are shaded *red* to *blue*, respectively, and scaled per row (GSEA, FDR < 0.25). *Grey* indicates no significant enrichment (GSEA, FDR ≥ 0.25)

Analysis of these genes in Blast2GO revealed numerous gene ontology terms that were enriched in each life stage transition (Additional file 2). Using GSEA on all genes, we found 86 KEGG pathways enriched in at least one transition (Fig. 5) and 53 pathways enriched in two or more transitions. Results largely agreed with those found in Blast2GO, even though these methods rely on different annotation, analysis, and tests for significance. Because GSEA uses only those transcripts that can be annotated to HGNC and combines expression of transcript variants to show gene-level transcription, we also examined aging-related KEGG-defined pathways using annotation defined by refseq and swissprot.

There were 6251 genes with significant differential expression in one or more sequential life stages. Here we summarize our analyses of each life stage transition, with emphasis on gene expression changes involved in specific processes related to aging, in particular the shift from young, reproductive females to aged, senescent, post-reproductive females.

### Eggs to neonates

Almost 10% of the transcriptome (2028 genes) is differentially expressed in the transition from egg to neonate (Figs 2, 3 and 4). Enriched GO terms and KEGG pathways are characteristic of a switch from a developing embryo to a swimming, feeding animal.

Nearly two thirds (715/1152) of genes that significantly decrease expression in this transition, many by more than 2^5^ (5 fold), do not increase expression at any later stage, indicating that they are highly expressed only in eggs and suggesting that they are specific to embryonic development. Consistent with this interpretation, these genes are involved in development, reflected in the down-regulation of GO terms including embryonic morphogenesis, organ morphogenesis, junction assembly, axon guidance, chemotaxis, and epithelium morphogenesis. The chitin-containing lorica and mastax of the rotifer are formed in the egg, and mastax size does not change after hatching, leading to the down-regulation of chitin catabolic processes after development (Additional file 2) [37].

Among all differentially expressed genes in this transition, 47% (413/876) of those that are up-regulated are later down-regulated, and 38% (434/1152) of those that are down-regulated are up-regulated after the neonate stage, suggesting that these genes are specifically involved in development of the neonate. These genes are enriched in a relatively small number of GO terms and KEGG pathways, and are primarily integral membrane proteins including ABC transporters, solute carriers, and cytochrome P450s, as well as notch1, and phospholipase B1, glutathione synthetase, and alkaline phosphatase (Additional file 2, Fig. 5).

More than half of genes with increased expression (455/876) do not decrease expression at any later stage and thus are specific to hatched animals rather than eggs. These genes are involved in metabolism, digestion, processing and excretion of waste, respiration, and sensing of the external environment.

### Neonates to early reproduction

The transition from the neonate to early reproductive stage is accompanied by the greatest change in the transcriptome, with 25% (5504) of examined genes significantly up- or down-regulated and 30% of up-regulated genes increasing by more than 2–5 fold (Figs 2, 3 and 4). However, only 15% of up-regulated genes (386/2718) and even fewer down-regulated genes (77/2786) are down-regulated or up-regulated in later stages, respectively, indicating that relatively few of these genes are specific to early reproduction and are instead likely responsible for the transition from developmental to reproductive stages. Environmental interventions or genetic changes that modify expression of these genes may alter the timing of entry into adulthood and thus impact both healthspan and lifespan.

Pathways down-regulated in the transition from neonates to early reproduction include those involved in signaling and nervous system function, including ABC transporters, GnRH signaling, long-term potentiation, and neuroactive ligand receptor interaction (Fig. 5). There is additional down-regulation of pathways involved in calcium-mediated muscle contraction and relaxation, including a decrease in expression of ATP2A2, tropomyosin and subunits of the troponin complex, actin, tintin, myosin light chain kinase, and voltage-dependent calcium channels. Other GO terms enriched in down-regulated genes include cell signaling and cell transport-neuro peptide Y receptor, G-protein coupled amine receptor, potassium channel, transmembrane transport and transport of organic anions, monamines, and sodium ions; macropinocytosis, an endocytic process, and monamine transport (Additional file 2). Taken together, these suggest developmental processes necessary for interaction with the environment, including gravitaxis, phagocytosis, and detection of abiotic or external stimuli, are completed during the neonatal stage.

The suite of genes up-regulated in the transition from neonates to early reproductive females appears to be driven largely by the onset of reproduction (Fig. 5, Additional file 2). Enriched pathways are dominated by the cell cycle (including positive and negative regulation of ubiquitin-protein ligase activity, cell cycle checkpoint, and regulation of mitosis); DNA replication (positive and negative regulation, leading and lagging stand elongation), recombination, and repair (ligase activity, DSB processing, homologous recombination, BER, NER, MMR); oogenesis; and maturation and cell division. Up-regulation of RNA polymerase, the spliceosome, and basal transcription factors suggest a general increase in transcription, while increases in cell cycle pathways, p53, and TGF-beta are expected to control cell growth, differentiation, apoptosis, and cellular homeostasis during growth and embryonic development.

### Early to late reproduction

There are only 236 genes up-regulated in the transition to late reproduction; of these 104 are not differentially expressed in earlier transitions, and nearly all (224) are not differentially expressed in the transition to reproductive senescence (Figs 2, 3 and 4). Most of those pathways up-regulated in the early to late reproductive transition—primarily those involved in metabolism—were also up-regulated in either or both of the earlier transitions. An exception is carbohydrate metabolic processes, up-regulated only in this transition (Additional file 2). In contrast, 1140 genes are down-regulated, 504 of which are not differentially expressed at any other stage. GO terms associated with reproduction, development, cell migration and organ morphogenesis all declined (Additional file 2), but only the leukocyte transendothelial migration pathway was significantly down-regulated in GSEA (Fig. 5). This down-regulation was due to the decreased expression of Ras family genes, including ROCK1, RAC2, RAC1, RAP1B, and RHOA MYL9, ITK, and PRKCs. These genes are involved in cell growth, differentiation, and survival. As rotifers are eutelic, one would expect these cell division and differentiation genes to be down-regulated after development and with the decline in reproduction during this period. Decreases in subunits of cytochrome B-245, a component of the microbicidal oxidase system of phagocytes and of ITK may signal a decreased capacity to fight off infection with increasing age. Declines in the expression of MYL9, MYL12B, ACTB, ACTG and PRKCs suggest a breakdown in cell regulation.

### Late reproduction to post reproduction

The transition to post-reproductive senescence has the smallest number of significantly differentially expressed transcripts (Figs 2, 3, and 4). Of the 53 up-regulated and 25 down-regulated genes, 35 and nine, respectively, are not differentially expressed in any other transition. Of these, 21 transcripts are unannotated, and are targets for further investigation. Annotated transcripts are involved in aerobic respiration, signaling, and transport (Additional file 3).

In order to understand how more subtle changes in transcription level of individual genes could result in significant changes in genetic pathways, we focused on pathways identified as enriched by GSEA. Examining the core genes in pathways and whether they are differentially expressed in multiple related pathways can reveal genes likely to have a disproportionate impact on aging.

Consistent with clustering of transcriptional profiles (Fig. 2) we identified multiple pathways differentially expressed only in the late- to post-reproductive transition. Pathways down-regulated in this transition but not enriched in any other include biosynthesis of unsaturated fatty acids, tubule reclamation, glutathione metabolism, insulin signaling, inositol phosphate metabolism, and purine metabolism (Fig. 5).

**Fig. 6:**
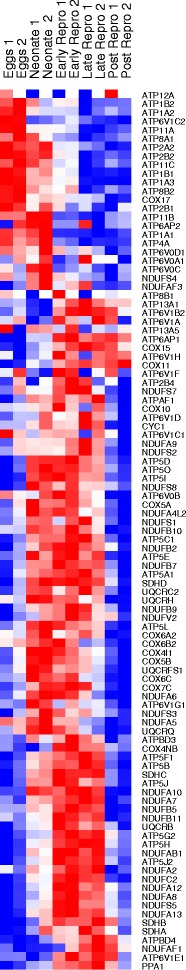
Relative expression of oxidative phosphorylation pathway genes between life stages in *Brachionus manjavacas*. Heatmap shows two biological replicates for each life stage. Data are row-normalized, with *red* indicating highest expression and *blue* indicating lowest expression

Pathways up regulated in one or more of the first three transitions and down-regulated in the transition to reproductive senescence were primarily in metabolism, maintenance and repair, and proteostasis (Fig. 5). Many of these seemingly distinct pathways, including those implicated in the age-related diseases of Parkinson’s, Huntington’s, and Alzheimer’s, were united by genes involved in mitochondrial function and oxidative phosphorylation, including NDUFA, UQCRC, COX genes, CYC1, SDHA, VDAC2 and SLC25A31, all components of the respiratory chain (Fig. 6). Declines in expression in these same pathways with aging appear to be evolutionarily conserved across a range of taxa [1, 38–40].

#### Metabolism

GSEA revealed a suite of metabolic pathways down-regulated in aged rotifers, including fatty acid, pentose phosphate, porphyrin and ether lipid metabolism, glycolysis, and the TCA cycle (Fig. 5). Multiple dehydrogenases were among the genes with the largest decreases in expression, including aldehyde dehydrogenases, hydroxyacyl-CoA dehydrogenase (HADH), malate dehydrogenases (MDH2, MDH1), and pyruvate dehydrogenase (PDHA1, PDHB). Phospholypases (PLAG2E, PLA2G3), aldolases (ALDOA, ALDOC), phosphoenolpyruvate carboxykinase 1 (PCK1), and pyruvate kinase (PKM2) also all decreased in the transition from late- to post reproduction (Additional file 4).

**Fig. 7:**
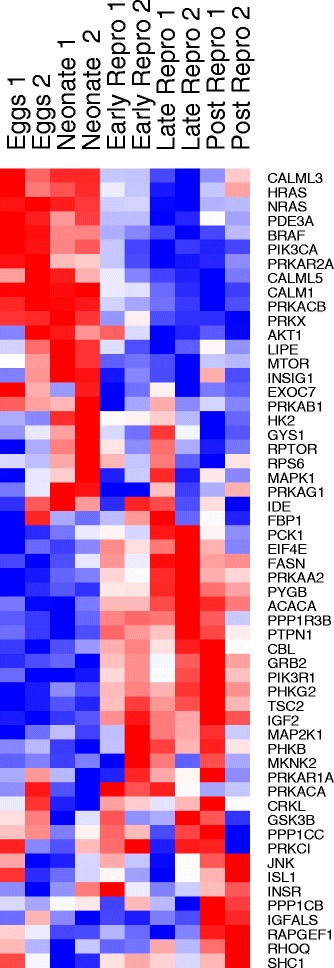
Relative expression of insulin signaling pathway genes between life stages in *Brachionus manjavacas*.. Heatmap shows two biological replicates for each life stage. Data are row-normalized, with *red* indicating highest expression and *blue* indicating lowest expression

Important signaling pathways with roles in metabolism, nutrient sensing, and oxidative phosphorylation were also down-regulated in post reproduction, most notably insulin, tor, and adipocytokine signaling. These pathways are known to play an important role in aging, and are effectors in increases in longevity in response to metabolism-related therapies, including caloric restriction [38, 41]. Expression of key aging-related genes, including JNK, INSR, IGF, IGFALS, and TOR—RNAi knockdown of which is known to increase lifespan in rotifers and other animal models [25, 42–45]—all increase slightly in senescence (Fig. 7). Other components of the insulin signaling and TOR pathways, particularly calcium-signaling genes, exhibit decreases in expression late in life. Together, these data suggest a synchronized breakdown in metabolic function and capacity late in life.

#### Proteostasis

**Fig. 8:**
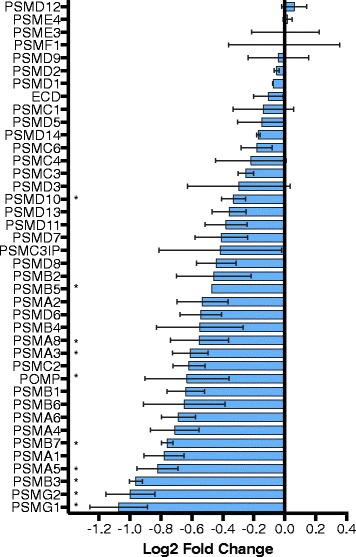
Expression change of proteasome subunit and assembly genes in the late- to post reproduction transition. Bars show mean fold change ± standard deviation of two biological replicates. * indicates significantly different expression between the two life stages (*p* ≤ 0.05, one-tailed *t*-test)

A decline in protein homeostasis is considered one of the hallmarks of aging across taxa [1], and our results suggest such a decline in late life in *B. manjavacas*. Expression of nearly all proteasome-related genes increases steadily over life until the transition to reproductive senescence, when expression of 31 out of 38 annotated structural proteasome subunit and catalytic co-factor genes decreased by up to 2.4-fold (Fig. 8); GSEA results capture the late-life decline in proteasome expression (Fig. 5). The proteasome degrades damaged or short-lived endogenous proteins, particularly cyclins and transcription factors, and recycles amino acids to be used in synthesis of new proteins. Proteasome function is thus essential for many cellular processes, including the regulation of gene expression, the cell cycle, and the response to cellular stress. A decline in the proteasome leads to an accumulation of mis-folded and aggregated proteins resulting in proteotoxic stress. A loss in proteostasis, aggregation of proteins, and widespread changes in the proteome are associated with aging in *C. elegans* [46, 47]. Interestingly, expression of the ubiquinated proteolysis pathway, which targets damaged proteins for removal via the proteasome, did not change significantly over lifespan in rotifers, suggesting that globally, ubiquitination is not the rate-limiting step in proteasomal degradation in late age, though declines in ubiquitination of specific targets likely play a role in aging.

Protein metabolism pathways were generally down-regulated in post-reproductive rotifers (Fig. 5). Leading edge analysis revealed the subset of genes that were commonly down-regulated between multiple protein metabolism pathways (Additional file 4). These were generally involved in catalyzing formation and then breakdown of S-adenosylmethionine (MAT1A, MAT2A, AHCY, CTH, and WBSCR22), ultimately decreasing the production of methyl groups available for methylation. Expression of the protein methyltransferases METTL6, METTL2B, and LCMT2 also declined. A suite of dehydrogenase enzymes that function in the mitochondria were down-regulated (GCDH, ALDH3A2, ALDH2, HADH, HADHA, ALDH4A1, GLUD1), affecting most protein metabolism pathways and linking amino acid metabolism and degradation with the butanoate and propanoate metabolism pathways.

**Fig. 9:**
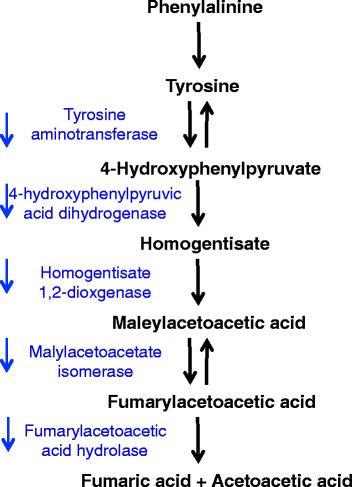
Expression change of tyrosine metabolism pathway genes between late- and post reproduction in *Brachionus manjavacas. Blue arrows* indicate the observed decline in expression of enzymes that catalyze metabolism of tyrosine

Expression of all the enzymes involved in the highly conserved, five-step tyrosine degradation pathway declined in post-reproductive rotifers (Fig. 9). Tyrosine is an important regulator of larval development and adult longevity in *C. elegans,* and acts as a signaling molecule involved in cell differentiation, growth, and maintenance [48]. Expression of homogentisate 1,2-dioxygenase (HGD) doubles in the transition from eggs to neonates, decreases slightly in the transition to early reproduction, doubles again in late reproduction, and declines again to less than half of previous levels in post-reproductive females. HGD is involved in the catabolism of tyrosine and phenylalanine and is best known as the autosomal recessive cause of alkaptonuria when mutated. Decreased function of HGD leads to a damaging buildup of homogentisic acid in connective tissues, and the inability to recycle phenylalanine and tyrosine into new proteins [49]. Increases in tyrosine concentration or changes in post-translational modification are associated with age-related disease including cancer, diabetes, neurodegeneration, and cataracts [48, 50]. Tyrosine metabolism is tied with metabolism and signaling, and is actively controlled by insulin signaling and in turn tyrosine aminotransferase (TAT), which catalyzes the conversion of tyrosine to fumarate and acetoacetate, appears to modify the effects of *daf2/*IGFR through FOXO and AMPK, thus strongly tying metabolic and protein sensing, signaling, and regulation.

Cathepsin C (CC; dipeptidyl peptidase-1) expression significantly increased 2.2 fold in senescent rotifers (baySeq, *p* < 0.05). CC is a lysosomal cysteine protease that activates granule serine peptidases in inflammatory cells. Increases in CC are associated with inflammatory diseases, and CC protein levels are increased in the central nervous system in aging mice, contributing to pathogenic inflammation [51]. Across taxa, aging is correlated with chronic inflammation that is associated with disease, decline in physical function, and mortality; this remains an active area of investigation in the biology of aging [52, 53].

#### Signaling and interaction with the environment

**Fig. 10:**
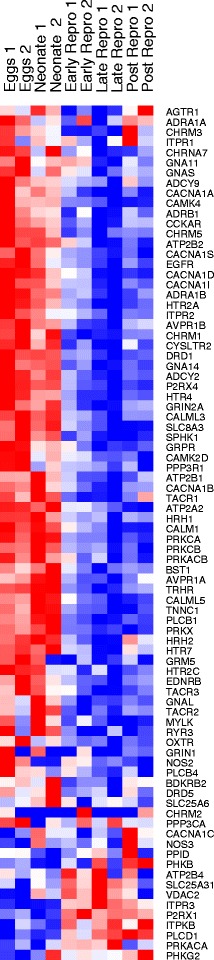
Expression change of calcium signaling pathway genes between life stages in *Brachionus manjavacas.* Heatmap shows two biological replicates for each life stage. Data are row-normalized, with *red* indicating highest expression and *blue* indicating lowest expression

The calcium pathway shows a dramatic shift over lifespan in rotifers, with the highest expression across nearly all calcium pathway related genes in eggs and neonates, a dramatic decrease during early and late reproduction, then a slight rise in expression of a subset of genes in the post-reproductive period (Fig. 10, Additional file 5). Several kinases involved in calcium signaling, including PhKG2, PRKACA and ITPKB also increase in late life. In contrast, there is decreased expression of SERCA (ATP2A2) calcium pumps in the endoplasmic reticulum, which could decrease cellular calcium buffering capacity, as found in aged neurons [54]. In rotifers, as in other animals, mitochondria appear to have a decreased ability to take up calcium in late life [55, 56].

The overall effect of the observed changes in gene expression is likely to be dysregulation of calcium homeostasis in senescent rotifers, leading to an increased intracellular calcium load that may negatively impact neuronal excitability [57–59]. In other model systems, loss of calcium homeostasis has been shown to be common to several age-related neurodegenerative diseases, including Alzheimers, Parkinson’s, and ALS [54, 60, 61]. The dysregulation of signaling and of sensing pathways, including neuron recognition and chemotaxis, beginning as soon as the early-reproductive period should be investigated further. Loss of the ability to sense and react appropriately to environmental conditions would be detrimental in late life.

### Epigenetic control of transcription across all life stages

**Fig. 11:**
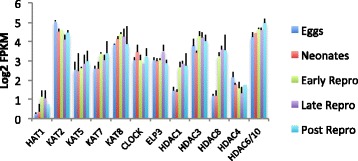
Expression of histone acetylase and deacetylase genes at each life stage in *Brachionus manjavacas.* Bars show mean expression as log2 of Fragments Per Kilobase of transcript per Million mapped reads (FPKM) ± standard deviation of two biological replicates

The level of 5-methylcytosine in *Brachionus* is below reliable limits of detection by LC-MS [62], and we and others have not found DNA methyltransferases (Dnmt1, Dnmt3) in the *B. manjavacas* transcriptome or in other published rotifer transcriptomes or genomes [15, 31, 62]. However, we report here that *B. manjavacas* has the molecular machinery for post-translational modifications to histone tails, including histone methylation and acetylation; these modifications play an important role in regulating gene expression. Most histone acetylases (HAT1, KAT5, KAT7, KAT8, CLOCK, ELP1) remained relatively constant with age, while histone deacetylases, activity of which is associated with repressed transcription, increased after the developmental period in eggs and neonates and remained high through late life (Fig. 11). Expression of HDAC6/10 was highest in post-reproductive rotifers. Only HDAC4 declined slightly but steadily from eggs to late reproduction.

**Fig. 12:**
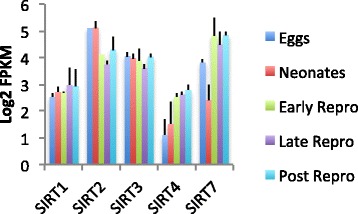
Expression of sirtuin family genes at each life stages in *Brachionus manjavacas*. Bars show mean expression as log2 of Fragments Per Kilobase of transcript per Million mapped reads (FPKM) ± standard deviation of two biological replicates

Sirtuin proteins are NAD+ dependent deacetylases, and are conserved regulators of aging and age-related diseases [63]. In *B. manjavacas* we found homologs of human sirtuins, based on amino acid sequence similarity: SIRT1 (nuclear), SIRT2 (cytoplasmic), SIRT3, SIRT4 (both mitochondrial) and SIRT7 (nuclear). Expression of SIRT1 and SIRT3 were relatively constant over rotifer lifespan (Fig. 12). Decrease in SIRT1 (the mammalian ortholog of yeast Sir2) by RNAi shortens lifespan in model organisms, while lifespan-extending caloric restriction up-regulates SIRT1. While SIRT4 expression was low, it rose 1.9-fold over the transitions from eggs to neonates to early reproduction. Sirt4 is a mitochondrial protein with additional activity as an NAD-dependent protein ADP-ribosyl transferase. SIRT2 expression, conversely, is high through development and early life, then declines by 1.1-fold through the reproductive and post-reproductive period. Sirt2 deacetylates a number of substrates, including H3K56, and H3K16, FOXO3, and alpha-tubulin [64–66]. Expression of SIRT7 displays the opposite profile, and is low in eggs and neonates then rises by 2.7-fold to remain high for the rest of life. SIRT7 specifically mediates deacetylation of H3K18, and is directly linked to the control of gene expression, particularly of nuclear hormone receptors [67]. SIRT7 plays a role in oncogenic transformation by suppressing the expression of tumor suppressor genes, and in humans SIRT7 expression is significantly elevated in breast cancer and thyroid carcinoma [68, 69].

**Fig. 13:**
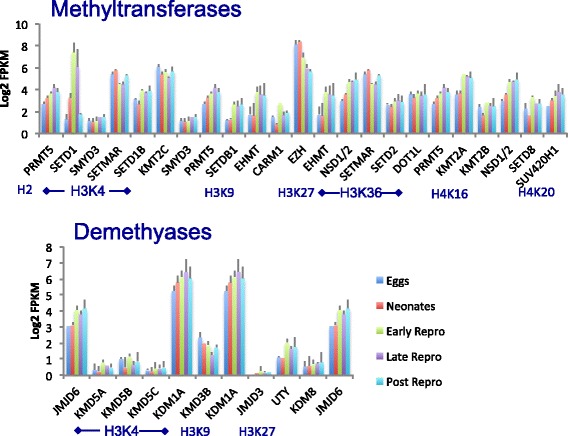
Expression of histone methyltransferase and demethylase genes at each life stages in *Brachionus manjavacas*. Bars show mean expression as log2 of Fragments Per Kilobase of transcript per Million mapped reads (FPKM) ± standard deviation of two biological replicates

Histone methyltransferases demonstrated a shift in expression between the first two (developmental) and last three (early-, late-, and post-reproductive) life stages (Fig. 13). In particular, expression of several genes that methylate H3K4, causing transcriptional activation, increased after development (KMT2A, 1.8-fold increase, KMT2B; 1.5-fold increase; SETD1B, 1.4-fold increase). Other, repressive histone methyltransferases also increased expression slightly in late life, including SETD8, SETD1B, EHMT, while expression of SUV420H1 decreased. The repressive polycomb group protein EZH, which methylates H3K9 and H3K27 and plays a key role in development and differentiation [70], declined in expression by 1.4-fold from neonates to early reproduction, and another 1.0-fold from early- to late reproduction.

There was little to no up-regulation of histone lysine demethylases in late age; only KDM1A, which demethylates H3K4 and H3K9, repressing transcription, increased 0.4-fold from early- to late reproduction, then declined by the same amount from late- to post reproduction. Expression of the H3R2 and H4R3 demethylase JMJD6 increased 1.1-fold between neonates and early reproduction, resulting in repression of transcription.

Together, these results suggest that large shifts in epigenetic markers may be partly or even largely responsible for driving the changes in gene expression over lifespan, an idea increasingly supported by results from other model systems [71, 72]. SETD1 and SETMAR provide particularly interesting targets for future investigation, as their expression changes greatly over lifespan, and not only in the transition from development to growth and reproduction. The increase (0.8-fold) of SETMAR and large decrease in SETD1A (5.2-fold) in the transition from late- to post reproduction suggests that they have functions beyond development, cell division, and differentiation.

### Differential expression during aging revealed in the rotifer model

Our assembly of the *B. manjavacas* transcriptome included 614 and 910 transcripts with potential homologs in the human genome absent in *D. melanogaster* and *C. elegans* genomes, respectively. Of these, 150 and 262 (~25%) were significantly differentially expressed in at least one life stage transition. Differentially expressed transcripts that lack homologs in both flies and worms correspond to 93 human genes, of which 20 are differentially expressed between eggs and neonates, 69 between neonates and early reproduction, 10 between early and late reproduction, and one between late reproduction and senescence (Additional file 6). In the egg to neonate transition, increased expression of arsenite methyltransferase (AS3MT), methionine adenosyltransferase 2B (MAT2B), and thiopurine S-methyltransferase (TPMT) enrich GO terms related to xenobiotic stimulus. In the neonate to early reproductive transition, increased expression of 12 genes enriched GO terms related to the cell cycle and DNA metabolic processes. Five of these and an additional three genes enrich GO terms associated with the microtubule organizing center and centrosome. The centrosome is involved in chromosome stability and has been suggested to play a role in aging [73–75].

## Conclusions

This study is the first to provide a detailed examination of the transcriptome and of changes in gene expression and gene pathways with normal aging in the rotifer *Brachionus manjavacas*, an emerging animal model for the study of the biology of aging. Shifts in expression in major aging related pathways very early in life suggest that changes in gene expression that cause or are caused by aging may actually begin at a relatively young age. We found a general decline in metabolism, signaling, proteostasis, and mitochondrial function associated with aging. Our findings highlight the important role in aging of changes in pathways that integrate many diverse functions, and suggest that subtle changes in expression of many genes, rather than a dramatic change in expression of individual genes, may lead to significant shifts in the function of biochemical pathways. A decline in pathways involved in cellular maintenance and repair such as the proteasome will lead to the accumulation of damage and prevent the recycling of cellular components. Signaling pathways such as calcium and insulin exert control across cellular and organismal processes as disparate as development, metabolism and neural function. The simultaneous declines in expression of energetic, signaling, and amino acid metabolism pathways are likely to have very different consequences for health and longevity than single or multi-gene knockdown in an otherwise healthy animal; thus investigation of subtle but genome-wide change in amino acid sensing and metabolism pathways during aging is an important area for future study. These results offer a framework that may be used to generate new hypotheses about the molecular genetic mechanisms of aging and provide a baseline against which to evaluate changes in gene expression due to interventions that change healthspan and lifespan.

## Additional files

Additional file 1: R script used for running baySeq. (TXT 4 kb)
Additional file 2: Gene Ontology terms significantly differentially regulated in sequential life history transitions (Blast2GO, FDR < 0.05). Categories: C = cellular component, F = molecular function, P = biological process. Worksheet 1: GO terms significantly up-regulated in neonates relative to eggs; Worksheet 2. GO terms significantly down-regulated in neonates relative to eggs; Worksheet 3: GO terms significantly up-regulated in early reproduction relative to neonates; Worksheet 4: GO terms significantly down-regulated in early reproduction relative to neonates; Worksheet 5: GO terms significantly up-regulated in late reproduction relative to early reproduction; Worksheet 6: GO terms significantly down-regulated in late reproduction relative to early reproduction. (XLSX 53 kb)
Additional file 3: Table of genes significantly differentially expressed in the late-to post-reproductive transition and at least one other transition, with KEGG description, and with charts showing changes in expression in genes significantly up (top) or down (bottom) regulated in the late- to post-reproductive transition. Forty-one unannotated genes were also up-regulated, and eight unannotated genes were down-regulated. (DOCX 143 kb)
Additional file 4: Leading edge analysis results for protein, carbohydrate, and fatty acid metabolism pathways. Worksheet 1: Change in gene expression in multiple protein metabolism pathways, as determined by leading edge analysis in GSEA. Shows those genes that are shared in more than one protein metabolism pathway (as denoted by X), not all genes in all pathways; Worksheet 2: Change in gene expression in multiple carbohydrate and fatty acid metabolism pathways, as determined by leading edge analysis in GSEA. Shows those genes that are shared in more than one protein metabolism pathway (as denoted by X), not all genes in all pathways. (XLSX 48 kb)
Additional file 5: The KEGG calcium signaling pathway showing genes up-regulated (red) and down-regulated (blue) in the late- to post-reproductive transition. (PNG 172 kb)
Additional file 6: Transcripts with potential orthologs in humans and not in *D. melanogaster* or *C. elegans*. (TXT 44 kb)

## Abbreviations

FPKM: Fragments mapped per kilobase of transcript per million reads
GO: Gene ontology
GSEA: Gene set enrichment analysis
HGNC: HUGO gene nomenclature committee
HUGO: Human genome organization
