# Supplementary material for: Genome-wide transcriptomics of aging in the rotifer Brachionus manjavacas, an emerging model system
Source: BMC Genomics. 2017 Mar 1;18:217. doi: 10.1186/s12864-017-3540-x (PMC5333405; doi:10.1186/s12864-017-3540-x)
Supplement: Additional file 3: — Table of genes significantly differentially expressed in the late-to post-reproductive transition and at least one other transition, with KEGG description, and with charts showing changes in expression in genes significantly up (top) or down (bottom) regulated in the late- to post-reproductive transition. Forty-one unannotated genes were also up-regulated, and eight unannotated genes were down-regulated. (DOCX 143 kb) [file 12864_2017_3540_MOESM3_ESM.docx]

| **Additional File 3. Differentially expressed genes shared with late-post transition:** | | | |
| --- | --- | --- | --- |
|  |  |  | |
| **Eggs-Neonates, Early-Mixed, Mixed-Post** | | | |
| **isotig** | **hit** | | **hitdesc** |
| contig00810 | K02256 | | cytochrome c oxidase subunit I [EC:1.9.3.1] |
| isotig00443 | K00412 | | ubiquinol-cytochrome c reductase cytochrome b subunit [ |
|  |  | |  |
| **Eggs-Neonates, Mixed-Post** | | | |
| isotig04228 | K14075 | | pancreatic lipase-related protein 2 [EC:3.1.1.3] |
| isotig12212 | K12383 | | ganglioside GM2 activator |
| ucontig9362 |  | | UA |
| ucontig31277 | | | UA |
|  |  | |  |
| **Eggs-Neonates, Neonates-Early, Early-Mixed, Mixed-Post** | | | |
| isotig04699 | K00451 | | homogentisate 1,2-dioxygenase [EC:1.13.11.5] |
| ucontig12544 | K05643 | | ATP-binding cassette, subfamily A (ABC1), member 3 |
| ucontig20566 | | | UA |
|  |  | |  |
| **Eggs-Neonates, Neonates-Early, Mixed-Post** | | | |
| ucontig40079 | K05119 | | anaplastic lymphoma kinase [EC:2.7.10.1] |
| isotig07136 | K11279 | | nucleosome assembly protein 1-like 1 |
| ucontig31612 | | | UA |
|  |  | |  |
| **Neonates-Early, Early-Mixed, Mixed-Post** | | | |
| ucontig9736 | K04558 | | synuclein, alpha interacting protein (synphilin) |
| isotig12926 | K08282 | | non-specific serine/threonine protein kinase |
| ucontig24015 | K04852 | | voltage-dependent calcium channel R type alpha-1E |
| isotig01630 |  | | UA |
| ucontig5972 |  | | UA |
| ucontig6350 |  | | UA |
| ucontig22448 | | | UA |
|  |  | |  |
| **Neonates-Early, Mixed-Post** | | | |
| isotig10303 | K07959 | | ADP-ribosylation factor-like 11 |
| ucontig10714 | K14388 | | solute carrier family 5 |
| isotig02210 | K06530 | | CD109 antigen |
| ucontig37215 | K01181 | | endo-1,4-beta-xylanase [EC:3.2.1.8] |
| ucontig34850 | | | UA |
| ucontig10571 | | | UA |
| ucontig16268 | | | UA |
| ucontig616 |  | | UA |
| isotig06264 |  | | UA |
|  |  | |  |
| **Early-Mixed, Mixed-Post** | | |  |
| isotig05623 |  | | UA |
| ucontig25774 | | | UA |
| ucontig38432 | | | UA |
| ucontig30650 | | | UA |
| ucontig6457 |  | | UA |
| ucontig15332 | | | UA |
| ucontig1811 |  | | UA |
| ucontig6972 |  | | UA |

UA: unannotated

Changes in expression in genes significantly up (top) or down (bottom) regulated in the late- to post-reproductive transition. Forty-one unannotated genes were also up-regulated, and eight unannotated genes were down-regulated.
