# Supplementary figures and images for: Genome-wide transcriptomics of aging in the rotifer Brachionus manjavacas, an emerging model system
Source: BMC Genomics. 2017 Mar 1;18:217. doi: 10.1186/s12864-017-3540-x (PMC5333405; doi:10.1186/s12864-017-3540-x)

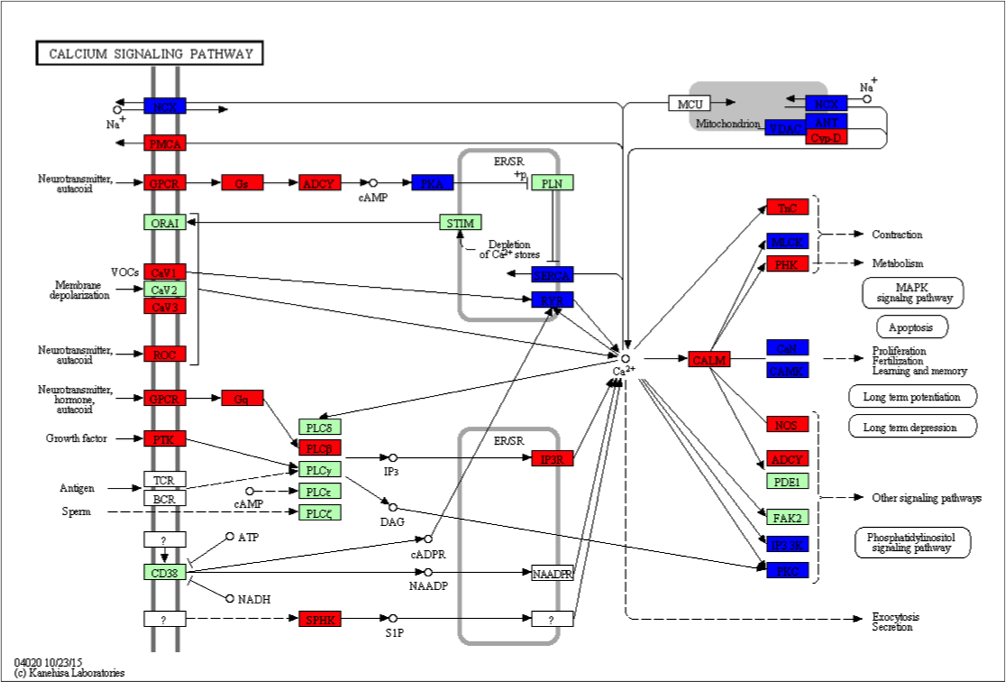

Supplement: Additional file 5: — The KEGG calcium signaling pathway showing genes up-regulated (red) and down-regulated (blue) in the late- to post-reproductive transition. (PNG 172 kb) [file 12864_2017_3540_MOESM5_ESM.png]
